# Comparison of Dietary Inorganic and Small-Peptide Chelating Trace Minerals on Growth Performance, Immunity, Meat Quality, and Environmental Release in *Litopenaeus vannamei*

**DOI:** 10.3390/ani15152297

**Published:** 2025-08-06

**Authors:** Jingshen Chen, Nan Liu, Shumeng Wang, Hailong Wang, Kun Ouyang, Yuxuan Wang, Junyi Luo, Jiajie Sun, Qianyun Xi, Yuping Sun, Yongguo Si, Yongliang Zhang, Ting Chen

**Affiliations:** 1 Guangdong Province Key Laboratory of Animal Nutritional Regulation, National Engineering Research Center for Breeding Swine Industry, State Key Laboratory of Livestock and Poultry Breeding, South China Agricultural University, College of Animal Science, Guangzhou 510642, China; 2 Key Laboratory of Animal Nutrition and Feed Science in South China, Ministry of Agriculture in Rural Affairs, Guangdong Key Laboratory of Animal Breeding and Nutrition, Institute of Animal Science, Guangdong Academy of Agricultural Sciences, Guangzhou 510640, China; 3 Liyang Aquatic Science and Technology Co., Ltd., Guangzhou 510510, China

**Keywords:** *Litopenaeus vannamei*, complex small peptide, trace minerals, growth, immunity, meat quality, environmental release

## Abstract

The present study evaluated the effect of adding 0% (control), 30%, 40% and 50% SPMs (small-peptide chelating trace minerals) to replace ITMs (inorganic trace minerals) in the diets of *Litopenaeus vannamei*; 720 shrimp were randomly assigned to four treatments (six replicates per group, 30 shrimp per replicate) in a 42-day feeding trial. There were no significant differences (*p* > 0.05) among the control, 40% SPM and 50% SPM groups in terms of the survival rate, weight gain rate, specific growth rate, hepatosomatic index, condition factor, feed intake, feed conversion ratio, or protein efficiency ratio; however, protein efficiency ratio was reduced in the 30% SPM group (*p* < 0.05). Glucose, triglyceride, and aspartate aminotransferase levels in the hemolymph of the 30% SPM group were significantly increased (*p* < 0.05), while the glucose and aspartate aminotransferase levels were also significantly increased in the 40% SPM group (*p* < 0.05). In the 50% SPM group, the glucose and triglyceride levels were also significantly increased (*p* < 0.05). Hepatopancreatic alkaline phosphatase activity was elevated at 40% SPM, and alkaline phosphatase, acid phosphatase, glutathione peroxidase, and total antioxidant capacity activities were significantly increased in the 50% SPM group (*p* < 0.05). The moisture content and drip loss were reduced in both the 40% and 50% SPM groups (*p* < 0.05). Therefore, replacing 40–50% ITMs with SPMs can maintain growth performance while enhancing physiological functions. In conclusion, the results of this study demonstrate that the incorporation of 30–50% SPMs into one’s diet constitutes a viable alternative to 100% ITMs.

## Simple Summary

ITMs, in their traditional form, exhibit suboptimal stability and low biological potency. Prolonged use of ITMs can result in *Litopenaeus vannamei* stress, significant environmental degradation, and potential food safety concerns. In contrast, SPMs boast a number of advantageous properties. SPMs possess a stable structure, high biological potency, rapid absorption, reduced mineral excretion, and an array of additional benefits. The incorporation of 50% SPM led to substantial enhancements in antioxidant capacity, meat quality, body coloration, and a reduction in fecal excretion of trace minerals in *Litopenaeus vannamei*. These findings suggest that SPMs can effectively replace ITMs at 40–50% levels in *Litopenaeus vannamei* diets, maintaining growth performance while enhancing physiological functions and reducing environmental impact.

## 1. Introduction

*Litopenaeus vannamei* (Boone, 1931), commonly known as the Pacific white shrimp, is an important penaeid species that is native to the eastern Pacific coast of South America [1]. This shrimp is characterized by its rapid growth rate and shortened production cycle, together with robust disease resistance, broad salinity tolerance, and high feed conversion efficiency [2]. Its superior flesh quality has rendered *Litopenaeus vannamei* the predominant species in large-scale, intensive aquaculture operations both worldwide and in China [3].

The success rate of *Litopenaeus vannamei* farming is generally low. This is due to changes in the environment and nutrition, as well as other serious stress reactions, resulting in a decline in immunity and the occurrence of diseases. These problems have become more frequent due to the expansion of aquaculture scale and the increase in its degree of intensification. The common diseases affecting *Litopenaeus vannamei* primarily include viral and bacterial diseases, as well as black gill disease. Presently, cultural practices are predominantly focused on prevention, encompassing both the mitigation of risk factors and the treatment of diseases. However, there is an absence of effective treatment options for viral diseases [2]. In addition to the implementation of proper culture management practices, the provision of high-quality, efficient shrimp feed that provides a comprehensive array of nutrients is crucial for ensuring the yield of *Litopenaeus vannamei* [4].

In the context of the intensive culture model, *Litopenaeus vannamei* exhibits a requirement for augmented intake and uptake of minerals from exogenous feeds and culture waters. Trace minerals (TMs) play a crucial role in the health and productivity of livestock, poultry, and aquaculture species. Not only do they participate in various physiological and metabolic processes, but they also promote healthy growth and improve overall production performance [5,6]. Common TMs, including Zn, Cu, Se, and Mn, are known to enhance animal immunity, improve feed conversion rates, and prevent or treat several nutritional deficiency diseases [7,8,9]. However, excessive use of these inorganic trace minerals (ITMs), particularly in intensive farming practices, can lead to significant environmental issues due to the high quantities discharged with feces [10,11]. Elevated levels of TMs in waste can result in the heavy metal pollution of soils, disrupt ecosystem balance, and adversely impact water quality and biodiversity [12,13]. Consequently, optimizing the use and management of TMs to mitigate their environmental impact has become an urgent issue in modern livestock and aquaculture practices.

In addition to environmental release, the bioavailability of TMs is also one of the problems faced by ITMs. Furthermore, excessive levels of TMs may compromise the activity of enzymes, vitamins, and other vital nutrients, ultimately interfering with the physiological functions and overall health of the animals [14,15,16,17]. In contrast, organic trace minerals (OTMs) have proven effective in reducing their detrimental effects on nutrients and nutritional antagonism [18]. Small-peptide chelating trace minerals (SPMs) are a form of new OTMs. SPMs have drawn plenty of attention due to their advantages in stability, absorptivity, and safety [19]. Moreover, the absorption utilization rate and biological potency of SPMs surpass those of ITMs. This superiority is attributed to the ability of small peptides to efficiently traverse intestinal mucosal cells and enter systemic circulation, resulting in rapid absorption and low energy consumption [20,21]. Additionally, chelated trace minerals (CTMs) offer enhanced support for physiological functions in animals, leading to improved immunity, growth promotion, and increased feed conversion rates [22,23]. Importantly, the use of CTMs can also reduce the heavy metal content in animal feces, thereby diminishing the potential for environmental pollution. Consequently, the environmental protection value of CTMs in the aquaculture industry has garnered increasing attention [16,24]. Thus, the application of these CTMs not only enhances livestock and poultry performance but also contributes to sustainable breeding practices, aligning with the ongoing trends in modern animal husbandry toward improving efficiency while ensuring environmental protection.

Organic zinc (zinc amino acid chelate) and organic copper (copper amino acid chelate) both boost growth and tissue trace element content, reduce free radicals and enhance antioxidants, increase lysozyme, immunoenzymes, and other enzyme activities in the hepatopancreas [25,26]. Organic magnesium (magnesium amino acid chelate) boosts growth and reduces oxidative stress [27]. Replacement of high doses of inorganic trace elements by low doses of hydroxyl methionine chelates of copper, zinc, and manganese did not affect the growth performance of *Litopenaeus vannamei*, whereas an appropriate increase in the level of hydroxy methionine chelate replacement could improve the antioxidant capacity of *Litopenaeus vannamei* and increase enzyme activities in vivo [28]. The mineral–amino acid complex (Zn, Mn, Cu, Fe, Se) is about 200% more effective than inorganic minerals for shrimp growth. The hemocyte count increased in the *Litopenaeus vannamei* fed the mineral–amino acid complex versus the inorganic mineral group. Lower drip loss in peeled *Litopenaeus vannamei* after 96 h with the mineral–amino acid complex was noted [29].

Notably, there is a lack of research on the application of SPMs in *Litopenaeus vannamei*. Therefore, the present study aims to investigate the effects of various concentrations (30%, 40%, and 50%) of SPMs on growth performance, immunity, meat quality, and the environmental release of TMs in *Litopenaeus vannamei*. This study was conducted to evaluate the practical significance of reducing the use of SPMs to improve the utilization of TMs in the diet of *Litopenaeus vannamei* while mitigating the environmental impacts associated with their excretion, and optimizing their nutritional management.

## 2. Materials and Methods

### 2.1. Diets and Experimental Design

The ITM and three SPM mixtures used in this study were provided by Guangdong Xingtengke Biotechnology Co., Ltd. (Zhaoqing, China). The experimental diet was provided by Liyang Aquatic Science and Technology Co., Ltd. (Guangzhou, China). The ingredients composition and proximate composition of the diets are presented in Table 1.

Healthy *Litopenaeus vannamei* were hatched from the same batch, of similar weight, with no injuries or diseases on the body surface, and were supplied by Liyang Aquatic Science and Technology Co., Ltd. (Guangzhou, China). The experiment was conducted at Liyang Aquatic Science and Technology Co., Ltd. (Guangzhou, China). A total of 720 *Litopenaeus vannamei* were randomly assigned to 4 groups (6 replicates per group, 30 shrimps per replicate) as follows: The control group (basal diet + ITM), 30% SPM group (providing 30% of the control group ITM level), 40% SPM group, and 50% SPM group. The experiment lasted for 42 days. Each dietary treatment is distributed into 6 black breeding drums, each with a capacity of 500 L. The initial daily feeding volume was 4% of the control body weight, and the shrimps were fed four times per day at 8:00, 13:00, 18:00, and 23:00. During the experiment, the feeding rate was adjusted according to the changes in growth status, feed intake, water temperature, and other factors. The seawater salinity was 30‰, the water temperature was maintained at 26–28 °C, and the pH was 7.2–7.5 throughout the culture period. Water was changed daily (30% during the early stage and 50% during the middle and late stages), and the oxygen was continuously oxygenated for 24 h, ensuring dissolved oxygen levels exceeded 7.0 mg/L.

### 2.2. Growth Performance and Body Indices

In the 6 weeks, after fasting the shrimp for 24 h, the growth parameters, including the survival rate (SR), specific growth rate (SGR), weight gain rate (WGR), feeding rate (FR), feed conversion ratio (FCR), protein efficiency ratio (PER), hepatosomatic index (HSI), and condition factor (CF), were calculated based on the recorded data. The formulae used were as follows:

Initial average body weight (IBW, g)

Final average body weight (FBW, g)

SR (%) = (Final shrimp number/Initial shrimp number) × 100

WGR (%) = (Final body weight—Initial body weight)/Initial body weight × 100

FCR = Dry feed intake (g)/Total weight gain (g)

FI(%/d) = Feed Consumption Dry Weight (g)/[(Initial Weight (g) + Final Weight (g)) × 0.5 × Number of days] × 100

SGR (%/d) = [(ln(Final body weight)—ln(Initial body weight))/Number of days] × 100

PER (%) = Total weight gain (g)/Dry protein intake (g) × 100

HSI (%) = Hepatopancreas weight (g)/Total body weight (g) × 100

CF (g/cm^3^) = [Body weight (g)/(Body length (cm))^3^] × 100

SR (%) = (Final number of individuals/Initial number of individuals) × 100

where FBW is the final body weight in g, IBW is the initial body weight in g, and the number of days is the experimental duration in days [34].

### 2.3. Sample Collection

In this experiment, *Litopenaeus vannamei* underwent a 7-day acclimatization period to the diet before the commencement of the 6-week feeding period. Starting from the fourth week, 4 replicates of each group were selected to collect feces and were stored in the refrigerator at −20 °C. At the end of the experiment, 4 shrimps were randomly selected from each replicate for hemolymph extraction (hemolymph to anticoagulant ratio 1:1). The extracted hemolymph was stored in a 1.5 mL centrifuge tube, centrifuged at 5000 r/min for 10 min at 4 °C, and the supernatant was stored in a refrigerator at −80 °C until testing. The shrimp were dissected immediately after blood collection, and the hepatopancreas was stored in the refrigerator at −80 °C until testing [35].

### 2.4. Measurement of Hemolymph Biochemical Parameters

Glucose (GLU), total protein (TP), albumin (ALB), triglyceride (TG), total cholesterol (T-CHO), aspartate aminotransferase (AST), and alanine aminotransferase (ALT) were measured using kits (F006-1-1, A045-2-2, A028-2-1, A110-1-1, A111-1-1, C010-2-1, and C009-2-1) provided by Nanjing Jiancheng Bioengineering Institute (Nanjing, China). The relevant indicators were measured, and the data were analyzed according to the instructions of the kit.

### 2.5. Measurement of Hemolymph and Hepatopancreas Antioxidant Indices

Precooled saline was added to the hepatopancreas according to the mass-to-volume ratio (*w*/*v*, 1/10), homogenized at 4 °C, centrifuged at 4000 r/min for 10 min at 4 °C, and the supernatant was collected. Alkaline phosphatase (ALP), acid phosphatase (ACP), total superoxide dismutase (T-SOD), glutathione peroxidase (GSH-Px), total antioxidant capacity (T-AOC), malondialdehyde (MDA), copper/zinc superoxide dismutase (Cu/Zn SOD), catalase (CAT), and ceruloplasmin (CP) in the hemolymph and hepatopancreas were measured using kits (A059-2-2, A060-2-2, A001-1-2, A005-1-2, A015-2-1, A003-1-2, A001-4-1, A007-2-1, and A029-1-1) provided by Nanjing Jiancheng Bioengineering Institute (Nanjing, China). The relevant indicators were measured, and the data were analyzed according to the instructions of the kit.

### 2.6. Routine Nutrient Composition Determination of Feed and Shrimp Meat

The moisture content was determined by the direct drying method (GB-5009.3-2016). The crude ash content was determined by high-temperature burning method (GB-5009.4-2016). Crude protein content of shrimp meat was determined by the Kjeldahl nitrogen determination method (GB-5009.5-2016). Crude lipid content of shrimp meat was determined by the Soxhlet extraction method (GB-5009.6-2016).

### 2.7. Determination of Meat Quality

At the end of the experiment, the meat yield of the shrimp, as well as the drip loss rate, freezing exudation rate, cooking loss rate, shear force, and pH value of the shrimp meat were measured. The determination method is as follows:

Meat yield (%) = shrimp meat weight/whole shrimp weight.

Drip loss: Record the shrimp meat weight at 0 h. After placing at 4 °C for 24 h, pat dry the surface moisture of the shrimp meat, weigh again, and record the shrimp meat weight after 24 h.

Drip loss rate (%) = (shrimp meat weight at 0 h—shrimp meat weight after 24 h)/shrimp meat weight at 0 h.

Freezing loss: Record the shrimp meat weight at 0 h. Place at −20 °C for 24 h, then remove and allow the temperature to rise to 2–3 °C at room temperature. Take the shrimp meat out of the sealed bag, leave it at room temperature for 3 min, pat dry the surface moisture, and weigh the shrimp meat (shrimp meat weight after 24 h).

Freezing exudate rate (%) = (shrimp meat weight at 0 h − shrimp meat weight after 24 h)/shrimp meat weight at 0 h.

Cooking loss: Place the shrimp meat in a sealed bag, immerse in a water bath at 70 °C for 5 min, and then remove it. After cooling, pat dry the surface moisture of the muscle and weigh the cooked meat.

Cooking loss rate (%) = (shrimp meat weight before cooking − cooked meat weight)/shrimp meat weight before cooking.

Shear force: Measure the shear force of the cooked shrimp meat using a shear force instrument (C-LM3B; Tenovo International Co., Ltd., Beijing, China).

pH value: The pH_1_ of the fresh shrimp samples and the pH_24_ after being stored at 4 °C for 24 h were measured using the portable pH meter (Testo 20; Testo SE & Co. KGaA, Titisee-Neustadt, Germany) [36,37,38].

### 2.8. Color Parameters

Cephalothorax and the second abdominal segments of shrimp were measured for color analysis using the WSC-S colorimeter (OPTO-STAR; Matthäus GmbH & Co. KG, Eckelsheim, Germany), including lightness (L*), redness (a*), and yellowness (b*) [39].

### 2.9. Whole Shrimp and Feces TM Analysis

Feces and whole shrimp samples were dried at 65 °C for 48 h, crushed, and passed through a 40-mesh sieve. The processed samples were then placed into sealed bags and stored in a freezer at −20 °C for later analysis. Approximately 1 g of the prepared solid sample was weighed and placed in a crucible. It was heated gently until it carbonized and was smokeless, and then it was transferred to a muffle furnace and ashed at 550 °C for 3 h. After cooling, the sample was removed and dissolved in an appropriate amount of hydrochloric acid solution. The solution was transferred to a 100 mL volumetric flask, and the inner container and lid were rinsed 2–3 times with a small amount of water. The rinsing liquid was combined in the volumetric flask and diluted to the mark with water, and then it was mixed thoroughly for use. The prepared solution was analyzed for Fe, Cu, Mn, and Zn contents using an atomic absorption spectrophotometer (AA-6880; Shimadzu Corporation, Kyoto, Japan).

### 2.10. Data Analysis

The data were presented as the mean ± SEM and subjected to GraphPad Prism 8.0.2. In order to assess the statistical assumptions, the data normality was tested using the Kolmogorov–Smirnov test, and the homogeneity of variances was assessed by Levene’s test. Assuming the validity of these assumptions, the data underwent analysis using a one-way ANOVA. A significance level of *p* < 0.05 was considered for determining statistically significant differences.

## 3. Results

### 3.1. Effects of Dietary ITM and SPM Supplementation on Growth Performance of Litopenaeus vannamei

To compare the effects of ITMs and reduced use of SPMs on the growth of *Litopenaeus vannamei*, we conducted an assessment of shrimp growth performance over a period of 42 days. As shown in Table 2, the results demonstrated that the 30%, 40%, and 50% SPM groups exhibited no significant differences in SR, WGR, SGR, HSI, CF, FI, and FCR in the 30%, 40%, and 50% compared to the ITM group (*p* > 0.05). For the FBW, the PER levels were significantly lower in the 30% SPM group compared to the ITM group, whereas no significant differences were observed in the 40% and 50% SPM groups.

### 3.2. Effects of Dietary ITM and SPM Supplementation on Biochemical and Antioxidant Indices

#### 3.2.1. Effects of Dietary Supplementation on Hemolymph Biochemical Indexes of *Litopenaeus vannamei*

As shown in Table 3, compared to the ITM group, the levels of GLU, TG, and AST in the hemolymph of the 30% SPM group were significantly increased (*p* < 0.05). Similarly, in the 40% SPM group, we observed significant increases in the GLU and AST levels (*p* < 0.05). Moreover, in the 50% SPM group, the GLU and TG levels were also significantly increased (*p* < 0.05). However, there was no significant difference in the levels of TP, ALB, T-CHO, and ALT (*p* > 0.05).

#### 3.2.2. Effects of Dietary Supplementation on Antioxidant Indices of *Litopenaeus vannamei*

To better understand the effects of reducing the dosage of trace minerals by using SPMs on the antioxidant indexes of the lymph of *Litopenaeus vannamei*. As shown in Table 4, compared to the ITM group, there were no significant differences in the levels of ALP, ACP, T-SOD, GSH-Px, T-AOC, MDA, Cu/Zn SOD, CAT, and CP among the SPM groups (*p* > 0.05).

#### 3.2.3. Effects of Dietary Supplementation on Antioxidant Indicators in Hepatopancreas of *Litopenaeus vannamei*

As shown in Table 5, compared to the ITM group, there were no significant differences in the activities of ALP, ACP, T-SOD, GSH-Px, T-AOC, MDA, Cu/Zn SOD, CAT, and CP in the hepatopancreas of the shrimp in the 30% SPM group (*p* > 0.05). In contrast, the 40% SPM group showed a significant increase in the activity of ALP (*p* < 0.05), and the 50% SPM group showed a significant increase in the activities of ALP, ACP, GSH-Px, and T-AOC (*p* < 0.05), while the other antioxidant indicators did not show significant differences (*p* > 0.05).

### 3.3. Effects of Dietary ITM and SPM Supplementation on Conventional Nutrient Composition of Meat of Litopenaeus vannamei

As shown in Table 6, compared to the ITM group, the moisture content of shrimp meat significantly decreased in the 40% and 50% SPM groups (*p* < 0.05). There were no significant differences in other conventional nutrient compositions in the SPM groups (*p* > 0.05).

### 3.4. Impact of Dietary ITM and SPM Supplementation on the Meat Quality of Litopenaeus vannamei

As shown in Table 7, compared to the ITM group, the pH_1_ significantly increased in all of the SPM groups (*p* < 0.05), while the water-holding capacity (drip loss rate) of the shrimp meat significantly decreased in the 40% and 50% SPM groups (*p* < 0.05). Additionally, there was a significant reduction in the shear force of shrimp meat in the 30% and 40% SPM groups (*p* < 0.05), and the cooking loss in the 50% SPM group also significantly decreased (*p* < 0.05). However, it is important to note that other quality parameters, including the meat yield rate, pH_24_, and freezing loss, did not show significant differences among the groups (*p* > 0.05).

### 3.5. Effects of Dietary ITM and SPM Supplementation on the Color Characteristics of Litopenaeus vannamei

To investigate the impact of SPMs on the coloration of *Litopenaeus vannamei*, we measured the L*, a*, and b* values of the carapace and abdominal shell both before and after cooking across different treatment groups. As shown in Table 8, before cooking, there were no significant differences in the L*, a*, and b* values of the carapace and abdominal shell among all groups (*p* > 0.05). As shown in Table 9, after cooking, the L*, a*, and b* values of the carapace, as well as the L* values of the abdominal shell, remained statistically similar across the treatment groups (*p* > 0.05). However, it is noteworthy that the b* values of the abdominal shell were significantly lower in the 30% SPM group compared to the ITM group (*p* < 0.05). Additionally, the a* values of the abdominal shell in the 30%, 40%, and 50% SPM groups were significantly higher than those observed in the ITM group (*p* < 0.05), with the 50% SPM group displaying the highest a* value.

### 3.6. Determination of Trace Mineral Uptake and Excretion in Litopenaeus vannamei

**Figure 1:**
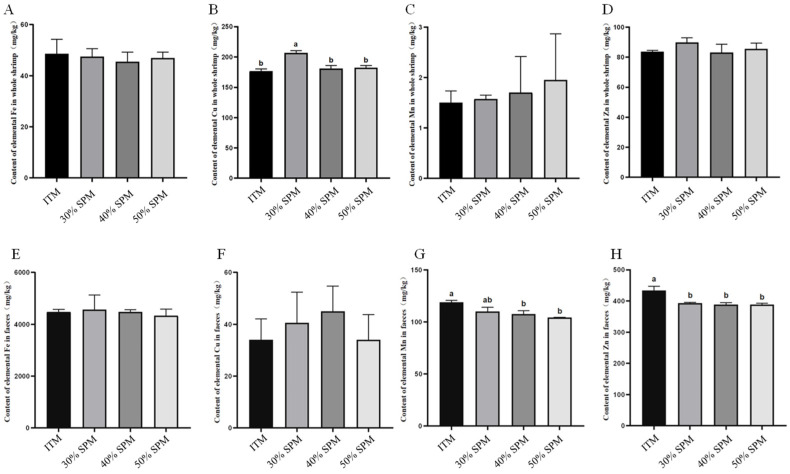
Effect of dietary ITM and SPM supplementation on trace minerals in whole shrimp (**A**–**D**) and feces (**E**–**H**), respectively. Different letters on bars (a, b) show a significant difference (*p* < 0.05). Values are presented as mean ± SEM of triplicate aquaria (*n* = 6).

To assess the uptake and excretion of trace minerals in shrimp, we measured the concentrations of trace minerals (Fe, Cu, Mn, and Zn) in both whole shrimp and their feces. Figure 1 shows that, compared to the ITM group, the 30% SPM group showed a significant increase in Cu content in whole shrimp (*p* < 0.05). In contrast, the 40% and 50% SPM groups did not exhibit significant differences in trace mineral concentrations compared to the ITM group (*p* > 0.05). Regarding fecal analysis, no significant differences in the Fe and Cu contents were observed among the 30%, 40%, and 50% SPM groups compared to the ITM group (*p* > 0.05). However, the Zn content in the feces was significantly reduced across all SPM groups (*p* < 0.05). Additionally, the Mn content in the feces of the 40% and 50% SPM groups was also significantly lower (*p* < 0.05). Notably, there were no significant differences in the Fe, Cu, Mn, or Zn concentrations among the SPM groups (*p* > 0.05).

## 4. Discussion

Growth performance is the most important indicator used to measure the economic benefits of animal production, and several studies have shown that trace minerals added in the form of organic chelates in feed can be absorbed more effectively, thereby maintaining animal growth performance [40,41,42]. In the broiler experiment, the ADG, ADFI, F/G, and mortality of broilers in the 30% and 50% OTM groups were comparable to those in the ITM group [43]. This is similar to another study conducted in broiler chickens that compared the reduced use of OTMs as a substitute for ITMs in maintaining broiler production performance [44]. In this study, the supplementation levels of 40% and 50% of OTMs had no significant effect on the growth indicators and food intake of *Litopenaeus vannamei*, indicating that the supplementation doses of 40% and 50% of SPMs had achieved the growth effect of 100% ITMs.

Blood biochemical indicators are typically associated with carbohydrate, lipid, and protein metabolism. These indicators provide a basis for evaluating an organism’s health and metabolic levels [45]. Total protein (TP) and albumin (ALB) are important indicators of health and immune function [46,47]. In this study, there were no significant differences in the TP and ALB levels in the lymph of the various groups. Primary indicators of carbohydrate and lipid metabolism in animals include blood glucose (GLU), triglycerides (TG), and total cholesterol (T-CHO). GLU is the primary energy source for animals. In this study, the GLU levels in the blood lymph of all three organic groups were significantly higher than in the inorganic group. This finding is consistent with a study on composite amino acid chelated trace elements in *Litopenaeus vannamei*, which indicates that replacing inorganic minerals with composite peptide-chelated trace elements can promote carbohydrate metabolism in *Litopenaeus vannamei* [28]. The TG and T-CHO levels in the hemolymph of the three organic groups increased, with the 50% SPM group having significantly higher TG levels than the ITMs and the 40% and 50% SPMs having increased TG and T-CHO levels. These changes may enhance the shrimp’s edible flavor.

Fe, Cu, Mn, and Zn are essential components of certain redox enzymes, maintaining the homeostasis of the body’s antioxidant system [48]. Adding appropriate amounts of trace minerals to the diet can significantly reduce serum MDA levels, while enhancing total antioxidant capacity and superoxide dismutase activity [49]. In studies on chickens, 30% and 50% OTMs were found to maintain serum T-AOC levels comparable to those in groups fed 100% ITMs [50]. Compared to 100% ITMs, supplementation with 37.5%, 50%, and 62.5% OTMs was able to maintain serum ALP levels in laying hens [51]. In this study, no significant differences were observed in various antioxidant indices in the hemolymph of shrimp fed diets containing 30%, 40%, and 50% SPMs. However, the ALP activity in the hepatopancreas of shrimp in the 40% SPM group was significantly increased, while the ALP, ACP, GSH-Px, and T-AOC activities in the hepatopancreas of shrimp in the 50% SPM group were significantly enhanced, potentially improving the antioxidant capacity of the shrimp.

Previous studies have suggested that OTMs enhance nutrient absorption and utilization, potentially altering the osmotic balance and water-holding capacity of muscle tissues [52,53]. The observed results indicate that the inclusion of 40% and 50% SPM groups significantly reduced the moisture content of shrimp muscle compared to the ITM group (*p* < 0.05). This reduction in moisture content may be attributed to the improved metabolic efficiency and mineral utilization associated with OTMs, which could influence water retention in muscle tissues [54,55]. The reduced drip loss in the 40% and 50% SPM groups (*p* < 0.05) indicates an improved water-holding capacity, which is crucial for maintaining meat quality during storage and processing; a higher bioavailability of organic minerals thereby affects the protein structure and muscle fiber properties [56]. The crude protein and crude fat content of the muscle tissue in the 40% and 50% SPM groups were slightly higher than in the control group, but these differences were not statistically significant. This suggests that SPMs can influence the nutritional composition of *Litopenaeus vannamei* muscle tissue, thereby improving meat quality. There was a trend towards an increased crude fat content in all three SPM groups, which correlates with the rise in blood lymph TG levels. This further supports the conclusion that composite peptide-chelated trace elements can influence lipid metabolism in *Litopenaeus vannamei*. In the experiment with broilers, the muscle shear force was significantly reduced in the OTM group compared with the ITM group [43]. In this study, the significant reduction in shear force values observed in 30% and 40% SPM groups (*p* < 0.05) suggests enhanced meat tenderness, while the decreased cooking loss in the 50% SPM group (*p* < 0.05) indicates better moisture retention during thermal processing. However, the lack of significant differences in meat yield, pH24, and thawing loss (*p* > 0.05) suggests that certain quality parameters remain stable regardless of mineral source. These findings are particularly valuable for aquaculture practices, as they demonstrate that lower inclusion levels of OTMs can effectively improve meat tenderness and overall meat quality, potentially offering both economic and quality benefits.

The coloration of crustaceans serves not only as a natural defense mechanism but also significantly influences the market value and consumer purchasing decisions in commercial species [57]. Notably, in this study, all SPM groups (30%, 40%, and 50%) exhibited significantly higher a* values in the abdomen compared to the ITM group after cooking (*p* < 0.05), with the 50% SPM group showing the highest redness intensity. This enhancement in red coloration could be explained by an improved absorption and utilization of dietary carotenoids, particularly astaxanthin, which is facilitated by OTMs [58]. These findings suggest that SPMs, especially at higher inclusion levels (50%), may enhance the development of desirable red coloration in cooked shrimp, potentially through improved pigment metabolism and stability.

Studies have shown that organic sources improve mineral absorption compared to inorganic trace minerals [59]. In the broiler experiment, organic Zn, Fe, and Mn were absorbed more efficiently in the body than inorganic Zn, Fe, and Mn [60,61,62]. In the pig experiment, the fecal Zn concentration during the nursery and fecal Cu concentrations during the growing and gilt-developer phases were lower in pigs that were fed the reduced chelated compared with the reduced inorganic treatment. And reducing the concentrations of Zn, Cu, Mn, and Fe typically supplemented to pig diets will greatly decrease fecal mineral excretion without negatively affecting pig performance from weaning through development [63]. Similar results showed that replacing 100% ITMs with 30–60% OTMs had no adverse effect on the average daily gain, average daily feed intake, feed/gain, carcass traits, or meat quality, while significantly reducing the contents of Cu, Zn, and Mn in feces [16]. In this study, the significant increase in whole-body Cu content observed in the 30% SPM group (*p* < 0.05) compared to the ITM group suggests enhanced Cu bioavailability at this level. The absence of significant differences in TM concentrations between the 40% and 50% SPM groups and the ITM group (*p* > 0.05) indicates that higher levels may not necessarily result in increased mineral retention. While no significant differences were observed in the fecal Fe and Cu contents across treatments (*p* > 0.05), all SPM groups demonstrated significantly reduced Zn excretion (*p* < 0.05). Additionally, the 40% and 50% SPM groups showed a significantly lower fecal Mn content (*p* < 0.05). These reduced mineral excretion levels suggest improved mineral utilization efficiency with organic sources.

## 5. Conclusions

In summary, dietary replacement of 30–50% ITMs with SPMs had no significant impact on the growth performance, feed utilization, or survival of *Litopenaeus vannamei*. The replacement level of 30–50% SPMs significantly enhanced the immune and antioxidant capacity of *Litopenaeus vannamei*, with elevated levels of key antioxidant enzymes in the hepatopancreas (ALP, ACP, GSH-Px, and T-AOC). The replacement level of 30–50% SPMs significantly improved the muscle quality and body color of *Litopenaeus vannamei*. The replacement level of 30–50% SPMs reduced the fecal excretion of trace elements and had no effect on whole shrimp trace element levels. Collectively, these results demonstrate that a dietary inclusion of 30–50% SPMs is a viable alternative to 100% ITMs, offering feed manufacturers and shrimp farmers a practical strategy to maintain feed efficiency while reducing mineral discharge into the aquatic environment.

## Figures and Tables

**Table 1:** Composition and proximate composition per kg of experimental diets.

Ingredients (As Is)	Diets			
	ITM	30% SPM	40% SPM	50% SPM
Fish meal (g/kg)	320.7	320.7	320.7	320.7
Meat meal (g/kg)	70.1	70.1	70.1	70.1
Soybean meal (g/kg)	220.4	220.4	220.4	220.4
Peanut meal (g/kg)	81.2	81.2	81.2	81.2
Wheat flour (g/kg)	220.4	220.4	220.4	220.4
Squid paste (g/kg)	30.1	30.1	30.1	30.1
Fish oil (g/kg)	10.0	10.0	10.0	10.0
Ca(H_2_PO_4_)_2_ (g/kg)	30.1	30.1	30.1	30.1
Choline chloride (g/kg)	2.0	2.0	2.0	2.0
Shrimp premix feed (g/kg) ^1^	10.0	10.0	10.0	10.0
Cr_2_O_3_	5.0	5.0	5.0	5.0
Total (g/kg)	1000	1000	1000	1000
FeSO_4_ (mg/kg)	140	0	0	0
CuSO_4_ (mg/kg)	15	0	0	0
MnSO_4_ (mg/kg)	30	0	0	0
ZnSO_4_ (mg/kg)	75	0	0	0
Peptide-chelated iron (mg/kg)	0	42	56	70
Peptide-chelated copper (mg/kg)	0	4.5	6	7.5
Peptide-chelated manganese (mg/kg)	0	9	12	15
Peptide-chelated zinc (mg/kg)	0	22.5	30	37.5
Proximate composition ^2^				
Moisture (g/kg)	39.3	38.1	33.8	33.4
Crude protein (g/kg)	426.1	428.7	428.5	425.8
Crude lipid (g/kg)	38.5	43.9	48.1	39.2
Crude ash (g/kg)	137.6	134.9	133.4	134.3
Lysine (g/kg)	24.6	24.6	24.6	24.6
Methionine (g/kg)	8.4	8.4	8.4	8.4
Cystine (g/kg)	4.5	4.5	4.5	4.5
Gross energy/(MJ/kg)	17.06	17.06	17.06	17.06

^1^ Sum of all ingredients = 1000 g/kg feed. The shrimp premix feed provides per kg of diet: vitamin A 6000 IU, vitamin D3 1500 IU, vitamin E 70.00 mg, vitamin K3 20.00 mg, vitamin B1 10.00 mg, vitamin B2 10.00 mg, vitamin B6 9.00 mg, vitamin B12 15.00 mg, nicotinamide 35.00 mg, folic acid 3.00 mg, D-Pantothenic acid 35.00 mg, D-biotin 0.03 mg, ferrum 140.00 mg, copper 15.00 mg, manganese 30.00 mg, zinc 75.00 mg, magnesium 100 mg, cobalt 0.25 mg, iodine 0.10 mg, selenium 0.30 mg, inositol 100.00 mg, and ethoxyquin 5.00 mg. ^2^ Crude protein, crude lipid, and crude ash are measured values; others are calculated values. The moisture content of the air-dried samples was determined by the direct drying method (GB-5009.3-2016) [30]. Crude ash was measured by high-temperature incineration (GB-5009.4-2016) [31]. Crude protein was quantified using the Kjeldahl nitrogen method (GB-5009.5-2016), and crude fat was extracted and measured via the Soxhlet extraction method (GB-5009.6-2016) [32,33].

**Table 2:** Effects of dietary ITM and SPM supplementation on the growth performance of *Litopenaeus vannamei*.

Items	ITM	30% SPM	40%SPM	50%SPM	*p*-Value
IBW	8.73 ± 0.29	8.55 ± 0.24	8.65 ± 0.26	8.66 ± 0.17	0.968
FBW	21.34 ± 0.24 ^a^	19.65 ± 0.30 ^b^	20.84 ± 0.17 ^a^	21.29 ± 0.29 ^a^	<0.001
SR (%)	98.29 ± 0.77	99.45 ± 0.56	98.37 ± 1.13	98.89 ± 0.70	0.734
WGR (%)	150.02 ± 7.59	148.95 ± 11.53	145.00 ± 9.02	147.65 ± 7.66	0.984
SGR (%/d)	2.15 ± 0.08	2.07 ± 0.12	2.01 ± 0.09	2.13 ± 0.08	0.679
HIS (%)	3.90 ± 0.13	4.31 ± 0.12	4.09 ± 0.22	4.23 ± 0.14	0.306
CF (g/cm^3^)	0.61 ± 0.01	0.60 ± 0.01	0.62 ± 0.02	0.60 ± 0.01	0.480
FI (%/d)	3.83 ± 0.07	3.85 ± 0.10	3.90 ± 0.09	3.88 ± 0.04	0.922
FCR (%)	1.85 ± 0.04	1.96 ± 0.07	1.94 ± 0.06	1.94 ± 0.07	0.534
PER (%)	1.23 ± 0.03 ^a^	1.10 ± 0.04 ^b^	1.16 ± 0.03 ^ab^	1.26 ± 0.02 ^a^	0.014

Values are presented as mean ± SEM of triplicate aquaria (*n* = 6). Values with different superscript letters within the same row are significantly different (*p* < 0.05).

**Table 3:** Effects of dietary ITM and SPM supplementation on hemolymph biochemical indexes of *Litopenaeus vannamei*.

Items	ITM	30% SPM	40% SPM	50% SPM	*p*-Value
GLU (mmol/L)	84.15 ± 2.81 ^b^	97.83 ± 1.96 ^a^	99.04 ± 2.92 ^a^	105.67 ± 4.09 ^a^	<0.001
TG (mmol/L)	0.61 ± 0.10 ^b^	0.94 ± 0.07 ^a^	0.73 ± 0.07 ^ab^	0.87 ± 0.07 ^a^	0.043
AST (U/L)	3.28 ± 0.54 ^b^	5.33 ± 0.35 ^a^	5.33 ± 0.23 ^a^	3.93 ± 0.15 ^b^	0.001
TP (g/L)	92.72 ± 10.92	91.57 ± 5.42	84.39 ± 6.07	93.66 ± 9.57	0.855
ALB (g/L)	19.49 ± 2.41	20.08 ± 1.06	19.23 ± 1.92	19.64 ± 2.47	0.992
T-CHO (mmol/L)	2.07 ± 0.22	2.44 ± 0.15	2.27 ± 0.16	2.46 ± 0.15	0.384
ALT (U/L)	16.88 ± 1.83	16.77 ± 1.89	17.37 ± 2.42	15.48 ± 1.40	0.909

Values are presented as mean ± SEM of triplicate aquaria (*n* = 6). Values with different superscript letters within the same row are significantly different (*p* < 0.05).

**Table 4:** Effects of dietary ITM and SPM supplementation on antioxidant indices of hemolymph of *Litopenaeus vannamei*.

Items	ITM	30% SPM	40% SPM	50% SPM	*p*-Value
ALP (U/L)	79.33 ± 5.39	87.68 ± 8.00	89.77 ± 11.50	98.13 ± 13.79	0.643
ACP (U/L)	481.45 ± 48.46	495.98 ± 66.41	390.61 ± 43.37	439.66 ± 50.09	0.506
T-SOD (U/mL)	206.37 ± 18.21	199.83 ± 11.49	187.69 ± 6.78	198.59 ± 15.65	0.842
GSH-Px (U/mL)	3141.82 ± 183.12	3300.78 ± 86.97	3123.12 ± 199.13	3263.38 ± 115.85	0.804
T-AOC (mmol/L)	0.45 ± 0.06	0.46 ± 0.02	0.50 ± 0.05	0.55 ± 0.04	0.484
MDA (nmol/mL)	6.55 ± 0.85	6.78 ± 0.60	6.90 ± 0.57	6.90 ± 0.57	0.980
Cu/Zn SOD (U/mL)	78.93 ± 6.17	70.58 ± 4.28	78.07 ± 4.85	76.26 ± 4.52	0.646
CAT (U/mL)	0.44 ± 0.01	0.34 ± 0.02	0.48 ± 0.10	0.57 ± 0.05	0.082
CP (U/L)	17.04 ± 2.84	15.21 ± 1.50	14.88 ± 1.67	19.01 ± 3.61	0.652

Values are presented as mean ± SEM of triplicate aquaria (*n* = 6).

**Table 5:** Effects of dietary ITM and SPM supplementation on antioxidant indices of hepatopancreas of *Litopenaeus vannamei*.

Items	ITM	30% SPM	40% SPM	50% SPM	*p*-Value
ALP (U/L)	13,400.33 ± 494.72 ^b^	13,153.63 ± 590.08 ^b^	15,452.52 ± 660.20 ^a^	15,753.83 ± 801.90 ^a^	0.020
ACP (U/L)	16,414.88 ± 1508.88 ^b^	14,620.76 ± 1275.53 ^b^	16,763.36 ± 1016.17 ^b^	22,476.50 ± 1497.03 ^a^	0.003
T-SOD (U/mL)	18.19 ± 0.62	17.38 ± 0.80	16.73 ± 0.98	15.45 ± 0.75	0.141
GSH-Px (U/mL)	535.76 ± 51.93^b^	628.86 ± 46.87 ^ab^	641.91 ± 45.33 ^ab^	737.62 ± 43.69 ^a^	0.057
T-AOC (mmol/L)	0.42 ± 0.01 ^b^	0.43 ± 0.04 ^b^	0.49 ± 0.04 ^ab^	0.57 ± 0.04 ^a^	0.043
MDA (nmol/mL)	3.76 ± 0.15	3.74 ± 0.20	3.72 ± 0.36	4.04 ± 0.10	0.730
Cu/Zn SOD (U/mL)	10.83 ± 0.42	8.59 ± 0.76	10.12 ± 0.61	9.97 ± 0.67	0.124
CAT (U/mL)	0.13 ± 0.01	0.13 ± 0.01	0.15 ± 0.01	0.14 ± 0.01	0.352
CP (U/L)	5.11 ± 0.81	6.77 ± 0.58	5.42 ± 0.95	5.91 ± 1.40	0.660

Values are presented as mean ± SEM of triplicate aquaria (*n* = 6). Values with different superscript letters within the same row are significantly different (*p* < 0.05).

**Table 6:** Effects of dietary ITM and SPM supplementation on conventional nutrient composition of *Litopenaeus vannamei* (air-dried basis).

Items	ITM	30% SPM	40% SPM	50% SPM	*p*-Value
Moisture (%)	75.52 ± 0.12 ^a^	75.49 ± 0.13 ^a^	75.10 ± 0.13 ^b^	74.96 ± 0.07 ^b^	0.018
Crude ash (%)	6.69 ± 0.08	6.59 ± 0.05	6.59 ± 0.06	6.58 ± 0.15	0.790
Crude protein (%)	85.34 ± 0.39	85.92 ± 0.22	86.06 ± 0.24	86.18 ± 0.17	0.197
Crude lipid (%)	1.04 ± 0.11	1.41 ± 0.06	1.30 ± 0.15	1.12 ± 0.09	0.090

Values are presented as mean ± SEM of triplicate aquaria (*n* = 6). Values with different superscript letters within the same row are significantly different (*p* < 0.05).

**Table 7:** Effects of dietary ITM and SPM supplementation on muscle quality of *Litopenaeus vannamei*.

Items	ITM	30% SPM	40% SPM	50% SPM	*p*-Value
pH_1_	7.34 ± 0.05 ^b^	7.49 ± 0.05 ^a^	7.50 ± 0.04 ^a^	7.47 ± 0.02 ^a^	0.044
Drop loss (%)	6.26 ± 1.03 ^a^	5.09 ± 0.06 ^ab^	4.66 ± 0.17 ^b^	4.65 ± 0.11 ^b^	0.053
Shear force (N)	19.11 ± 0.29 ^a^	15.92 ± 0.27 ^b^	16.10 ± 0.87 ^b^	16.94 ± 1.13 ^ab^	0.037
Cooking loss (%)	6.99 ± 1.10 ^a^	8.78 ± 0.39 ^a^	7.35 ± 0.73 ^a^	4.65 ± 0.29 ^b^	0.010
Meat yield rate (%)	50.65 ± 0.39	50.82 ± 0.60	50.04 ± 0.43	50.25 ± 0.61	0.703
pH_24_	6.89 ± 0.03	6.89 ± 0.02	6.88 ± 0.04	6.81 ± 0.04	0.316
Freezing loss (%)	2.75 ± 0.15	2.36 ± 0.15	2.37 ± 0.07	2.42 ± 0.21	0.278

Values are presented as mean ± SEM of triplicate aquaria (*n* = 6). Values with different superscript letters within the same row are significantly different (*p* < 0.05).

**Table 8:** Effects of dietary ITM and SPM supplementation on the body color of *Litopenaeus vannamei* (raw shrimp).

Items	ITM	30% SPM	40% SPM	50% SPM	*p*-Value
Carapace					
L*	33.97 ± 0.38	34.01 ± 0.46	33.80 ± 0.75	34.84 ± 0.99	0.772
a*	−1.43 ± 0.09	−1.37 ± 0.18	−1.39 ± 0.19	−1.49 ± 0.15	0.947
b*	5.98 ± 0.40	5.38 ± 0.67	5.15 ± 0.39	6.01 ± 0.54	0.553
Abdominal shell					
L*	33.20 ± 0.50	31.06 ± 0.63	32.81 ± 1.32	33.34 ± 0.53	0.213
a*	−2.06 ± 0.09	−1.46 ± 0.24	−2.06 ± 0.23	−2.34 ± 0.39	0.151
b*	3.66 ± 0.50	4.25 ± 0.84	3.85 ± 0.47	3.85 ± 1.16	0.963

Values are presented as mean ± SEM of triplicate aquaria (*n* = 6). Abbreviations: L*, lightness; a*, redness; b*, yellowness.

**Table 9:** Effects of dietary ITM and SPM supplementation on the body color of *Litopenaeus vannamei* (Cooked shrimp).

Items	ITM	30% SPM	40% SPM	50% SPM	*p*-Value
Carapace					
L*	64.44 ± 1.08	62.73 ± 0.90	61.01 ± 1.29	63.77 ± 1.33	0.772
a*	13.65 ± 1.52	13.31 ± 1.18	16.27 ± 1.26	16.54 ± 0.66	0.947
b*	23.75 ± 2.60	20.89 ± 1.78	26.13 ± 1.62	26.49 ± 0.61	0.553
Abdominal shell					
L*	72.81 ± 0.73	71.67 ± 1.44	72.34 ± 1.11	73.24 ± 0.57	0.213
a*	14.78 ± 0.76 ^b^	16.26 ± 0.19 ^a^	16.86 ± 0.36 ^a^	17.47 ± 0.28 ^a^	0.151
b*	31.49 ± 1.02 ^a^	27.91 ± 0.21 ^b^	30.48 ± 0.83 ^a^	30.12 ± 0.51 ^a^	0.963

Values are presented as mean ± SEM of triplicate aquaria (*n* = 6). Values with different superscript letters within the same row are significantly different (*p* < 0.05). Abbreviations: L*, lightness; a*, redness; b*, yellowness.

## Data Availability

The original contributions presented in the study are included in the article. Further inquiries may be directed to the corresponding author.
